# Developing a consensus research definition for profound autism using a modified Delphi method

**DOI:** 10.1186/s13229-026-00727-y

**Published:** 2026-06-30

**Authors:** Matthew Siegel, Catherine L. Lord, Helen Tager-Flusberg, Judith Ursitti, Alycia Halladay, Alison T. Singer

**Affiliations:** 1 Boston Children’s Hospital, Boston, MA USA; 2 Tufts University School of Medicine, Boston, MA USA; 3 Harvard Medical School, Boston, MA USA; 4 David Geffen School of Medicine, University of California, Los Angeles, USA; 5 Boston University, Boston, MA USA; 6 Profound Autism Alliance, Wellesley, MA USA; 7 Autism Science Foundation, New York, NY USA; 8 Rutgers University, Piscataway, NJ USA

**Keywords:** Autism spectrum disorder, Profound autism, Delphi process

## Abstract

**Background:**

The diagnostic term, autism spectrum disorder, encompasses the full range of impairments associated with the autism spectrum, a significant change from earlier DSM definitions that identified various subtypes of autism. However, the majority of autism-related research and cultural representations in recent years has focused on autistic individuals with strong language and cognitive abilities. In 2021, the administrative term Profound Autism (PA) was proposed by a Lancet Commission to draw attention to autistic people whose impairments require lifelong, round-the-clock care. The initial definition of PA included a minimum age and requiring 24/7 access to an adult to ensure safety, and suggested considering IQ, and verbal ability when defining this group. However, researchers have subsequently utilized widely varying criteria in studies of this group, producing results that are difficult to compare and limit the potential for the identification of this group to advance knowledge about their strengths and needs.

**Methods:**

To address the need for studies that use comparable samples and increase clarity in communication and interpretation of results, we carried out a systematic, multi-stage Delphi consensus study with over 70 participants who identified as researchers, caregivers, autistic individuals, and clinicians, to measure consensus on components of a research definition of PA. A series of questions was developed for the primary domains of interest.

**Results:**

After two rounds of review and voting, 76% of the respondents agreed on a research definition of PA of: adaptive functioning well below age level, requiring adult supervision to ensure physical and mental health, safety and well-being, being at least 8 years old, diagnosed with ASD, and having severely impaired cognitive abilities (reflected by IQ score below 50) and/or not verbally communicating other than single words or fixed phrases used predominantly to have their basic needs met.

**Limitations:**

Variations in access to measurement tools, as well as access to services across the world, limit the utility of this definition outside research practices, and a response rate of 58% yielded a final round sample size of 78 that may underrepresent portions of the stakeholder community and was overwhelmingly composed of respondents from the United States.

**Conclusions:**

Based on these findings, a new working research definition of profound autism is proposed. Adoption of this definition could improve comparability across research studies, enhance the impact and generalizability of results, and better target interventions and supports for this high-needs group.

**Supplementary Information:**

The online version contains supplementary material available at 10.1186/s13229-026-00727-y.

## Background

According to the current definition of autism spectrum disorder in the DSM-5, the diagnostic term encompasses the full range of the autism spectrum, levels of impairment, and co-morbidities [1]. This represented a significant change from earlier definitions in DSM III, III-R and IV in which various subtypes of autism were recognized and defined. In 2021, *The Lancet Commission on the Future of Clinical Care in Autism* proposed the administrative term Profound Autism (PA) to describe people with autism whose significant impairments require round-the-clock access to an adult. The report highlighted the need to distinguish this group of autistic individuals from those who were more able, with the hope that introducing the term “will spur both the clinical and research global communities to prioritize the needs of this vulnerable and underserved group” [2, p. 278].

The Lancet Commission provided an administrative definition of PA that included a minimum age of 8, requiring 24/7 care to ensure safety, and suggested this group would most likely have an IQ below 50, or be nonverbal or minimally verbal. The Centers for Disease Control followed up on this initial introduction of PA to investigate its prevalence among 8-year-old children, using data collected as part of the Autism and Developmental Disabilities Monitoring Network. The CDC operationalized the term by using a definition of: IQ below 50 or non/minimally verbal, which classified 26.7% of the autistic children with profound autism [3]. 

Autistic individuals with intellectual disability or who are non/minimally verbal have been repeatedly shown to have higher rates of medical and psychiatric comorbidities, serious externalizing behaviors, hospitalization, and lower adaptive functioning, adult employment and independent living [4–9]. It is also widely recognized in the field that the choice of interventions and supports provided to autistic individuals is largely determined by the individual’s speech and verbal and cognitive ability. Despite the wide range of challenges and needs, and stark differences in appropriate treatment approaches and outcomes, only a minority of behavioral or neuroimaging studies of autism have included profoundly autistic participants [10, 11]. The proportion of treatment studies that include severely affected autistic individuals has declined precipitously over the past two decades [10]. Inclusion of this group in autism research has been limited by a variety of factors, including stigma, perceived challenge, increased costs, researcher familiarity, a paucity of validated assessment tools, and barriers to access and participation for individuals and families, though several publications have identified best practices for enhancing inclusion in research [11, 12]. By recognizing this disparity and providing clear guidance on how profound autism should be defined in a research setting, the goal is to facilitate inclusion of these individuals in research and improve diagnosis, treatments, and supports across the lifespan for this underserved group. As an example of such an effort, there was an increase in research projects that included non-verbal autistic individuals following a NIDCD workshop and resulting papers in 2013 that worked to better define and operationalize assessment of this group [13]. 

Progress in a scientific field is critically dependent on rigorous and specific definitions with agreed-upon operationalization [14]. Since it was introduced in 2021, profound autism has been variously defined in research studies, producing a wide range of prevalence rates across the world based on ascertainment, diagnosis, and other factors (11–58%), difficulty in comparing datasets, and confusion in the field [4]. The lack of a consensus research definition has not only impeded research on profound autism but also has contributed to criticisms of considering profound autism as a distinct subgroup of the autism population [15]. In particular, the needs for interventions, precision medicines, community integration, employment and housing are being painted with a broad brush and not targeted to individual and group characteristics because the term “autism” has become so broad. The lack of a research definition obfuscates the need to develop specific interventions and supports, despite the widely held principle that treatment should be individualized. Whether or not one agrees with distinguishing profound autism from the rest of autism, research including these individuals, which represents a significant gap in our knowledge about autism, is significantly impacted by the lack of a consensus definition.

The goal of the current study was to develop a consensus research definition of profound autism, using a modified Delphi methodology [16, 17], grounded initially by the domains in the Lancet report, as determined by a broad group of researchers, clinicians, caregivers, and autistic individuals.

## Methods

**Fig. 1:**
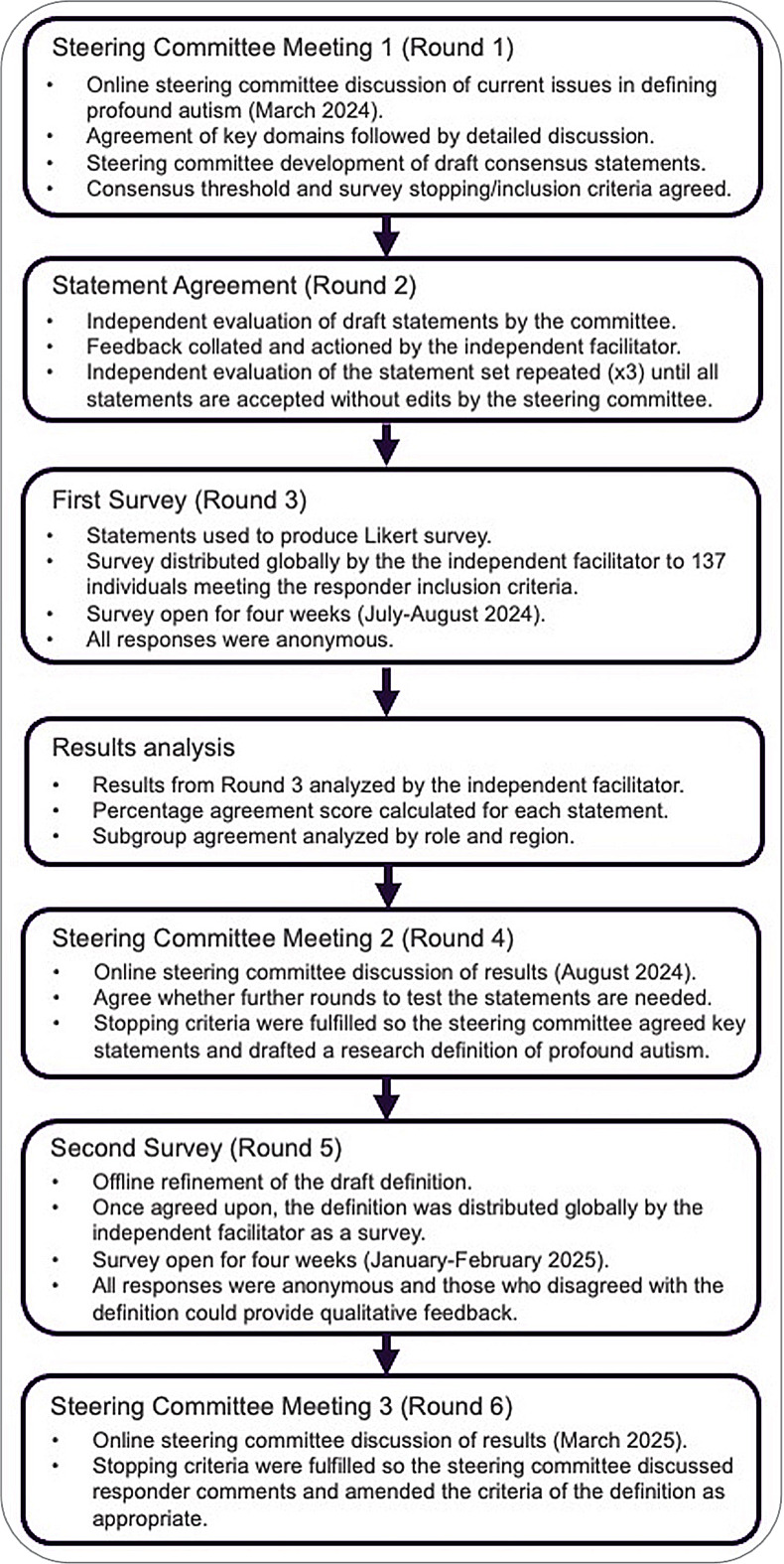
Modified Delphi process followed during the study

The study was carried out over the course of 2024–2025. It was led by a ‘steering committee’ (SC), which comprised the authors, guided by an independent facilitator with experience conducting Delphi research (Triducive Partners Limited). The study was not registered; all reporting follows the ACCORD guidelines [18]. Fig. 1 summarizes the modified Delphi process that was followed. The modified Delphi differs from a traditional Delphi in that the modified process is used to validate, refine or prioritize existing ideas rather than explore or build consensus from scratch. Specifically, the first rounds were not open ended, but started with a list of items based on the domains referred to in the previous Lancet paper. This resulted in fewer “rounds” being required. The surveys were anonymous.

### Ethics approval statement

This study did not require Institutional Review Board (IRB) approval because neither the assigned interventions nor the outcomes assessed were related to the health of participants. All survey respondents were informed of the purpose of the study and consent to participate was implied by the completion and submission of the survey. This research was conducted according to the principles of the Declaration of Helsinki.

### Developing domains and statements for inclusion in the survey

The SC convened in March 2024 and reached agreement on the key domains to be considered in developing a research definition of profound autism. Seven domains were drawn from those utilized in the Lancet paper, and an 8th category of “other” was included to allow for the addition of domains not included in the Lancet paper.

1. Age.
2. IQ.
3. Language/communication.
4. Adaptive functioning.
5. Need for adult support.
6. Co-morbid diagnoses.
7. Severe, intense, dangerous behavior.
8. Other.

Working collaboratively, the SC utilized these domains to develop an initial set of 35 statements with support from the facilitator. These statements underwent three rounds of review by members of the SC who categorized them as ‘accept’ ‘remove’ or ‘reword’. During the first round, 10 statements were removed, 7 were added, and 14 were edited. During the second round, 1 statement was removed, 1 was added, and 8 were edited. After a final review, 32 statements were accepted and developed into a survey. Each statement was presented with a 4-point Likert scale (strongly agree, tend to agree, tend to disagree, strongly disagree) to allow responders to indicate their level of agreement and to avoid the ambiguity which can be caused by including a middle option bias. Following standard Delphi methodology, this survey round was predominantly quantitative and did not seek feedback on the statements from survey respondents. Delphi is considered a consensus group research method, which involves obtaining the views of a group of experts and aims to achieve consensus and agreement as outcomes [19]. 

### Participants

The independent facilitator distributed the survey to a large group of individuals identified by members of the SC. They were identified by individual Steering Committee members as experts who were experienced clinicians, researchers, caregivers, or clinician-scientists whose expertise (published or presented at scientific meetings) included a focus on autistic individuals who were minimally verbal or had significant cognitive impairment. They were invited to participate and asked to self-identify on the basis of whether they were a researcher, clinician, caregiver, or an autistic individual. 137 individuals were invited to participate, 76 participated in round 1, and 70 participated in round 2. The great majority of those who agreed to participate were from the United States and only 8 represented 4 other geographic regions (Canada, Europe, Asia Pacific, and Middle East/Africa). The respondents came from diverse academic and professional backgrounds, suggesting that a comprehensive range of perspectives was included.

### Procedures

As this study only collected anonymous opinions and no identifying or patient health information, it was not submitted to an IRB. No incentives were provided to participants. A statement of consent was included at the beginning of the survey, and consent was assumed to be given by participant completion and submission of the survey. The only demographic information collected on the survey was the respondent’s primary self-identified role and geographic region.

The survey opened by asking participants if they were familiar with the term ‘profound autism’ and, in an effort to assess their understanding of the term, asking the open-ended question: “From your perspective, how is profound autism currently defined?” Participants then rated the 32 statements about Profound Autism using the Likert scale described earlier. Stopping criteria were established a priori as a one-month window within which participants could respond and a minimum of 70 responses.

**Fig. 2:**
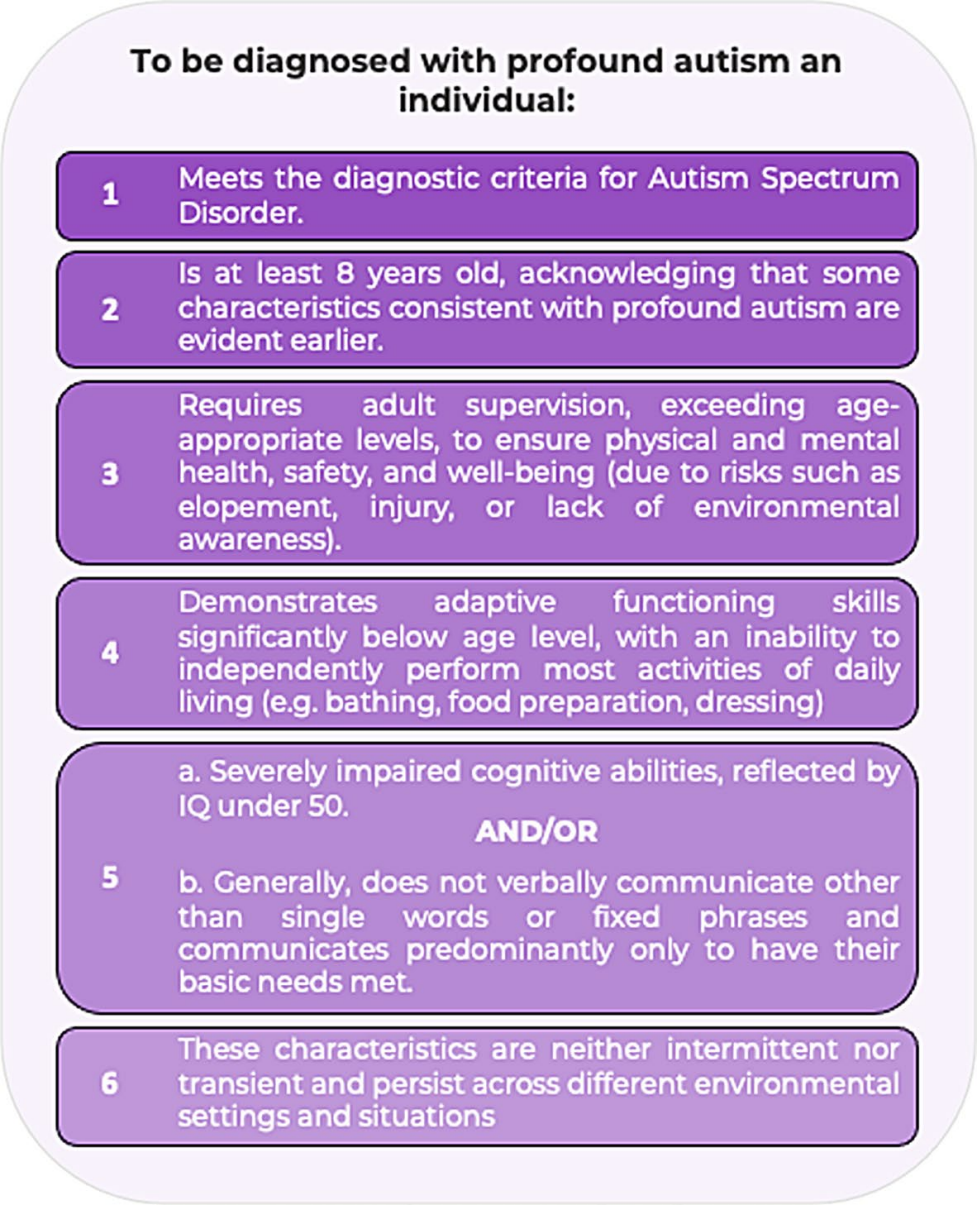
The finalized definition of profound autism based on the feedback from the second round of survey

Completed surveys were analyzed to determine an overall agreement level for each statement using descriptive statistics. The a priori threshold for consensus was set at 75% and then further defined as ‘strong’ (75%) or ‘very strong’ (90%). For Delphi consensus processes, a level between 70% and 80% is usually adopted and considered to be rigorous [20, 21]. The SC met in August 2024 to review the findings and, using the consensus level achieved, developed a research definition of PA. This definition, presented in Fig. 2, was tested in a second round of surveys, sent to the initial group of 76 responders to round 1.

The proposed definition was presented on the second-round survey, and participants were asked whether or not they agreed with it. If participants disagreed, they were asked to provide qualitative feedback on the definition. The same stopping criteria were used for the second-round survey.

## Results

### Initial survey

The initial survey was sent to 137 participants, and 70 responses were received, a response rate of 51% (see Supplementary Figure S1). Respondents were predominantly clinicians (*n* = 30, 45%), followed by researchers (*n* = 29, 41%), family members (*n* = 7, 10%), and autistic individuals (*n* = 2, 3%) and preferred not to say (*n* = 2). Most respondents (*n* = 62, 89%) were from the United States. Duplicates could be identified by unique identifying numbers assigned by the survey software based on the IP address. (Supplementary Figure S2).

The percentage of agreement with each of the 32 statements is presented in Table 1. There was very strong agreement (> 90%) with 12 statements, and strong agreement (> 75%) with an additional 8 statements. 12 statements failed to reach the consensus threshold, and were mostly clustered under the domains of IQ, age, and severe/dangerous behaviors.

### Second-round survey

The second-round survey was sent to the original 137 participants. It included a definition of profound autism based on the results from the first round and was completed by 78 participants (57%) (see Supplementary Figures S3 and S4 for demographic details). The majority of respondents agreed with the proposed definition (*N* = 59; 76%), exceeding the consensus threshold. The SC met one final time in December 2024 to discuss the feedback from the second-round survey and finalized the research definition of profound autism. The SC considered the high rate of agreement with the surveyed definition and the qualitative comments from some respondents that accompanied the ratings (see Supplementary Table 1) to make slight adjustments to the surveyed definition.

**Table 1:**
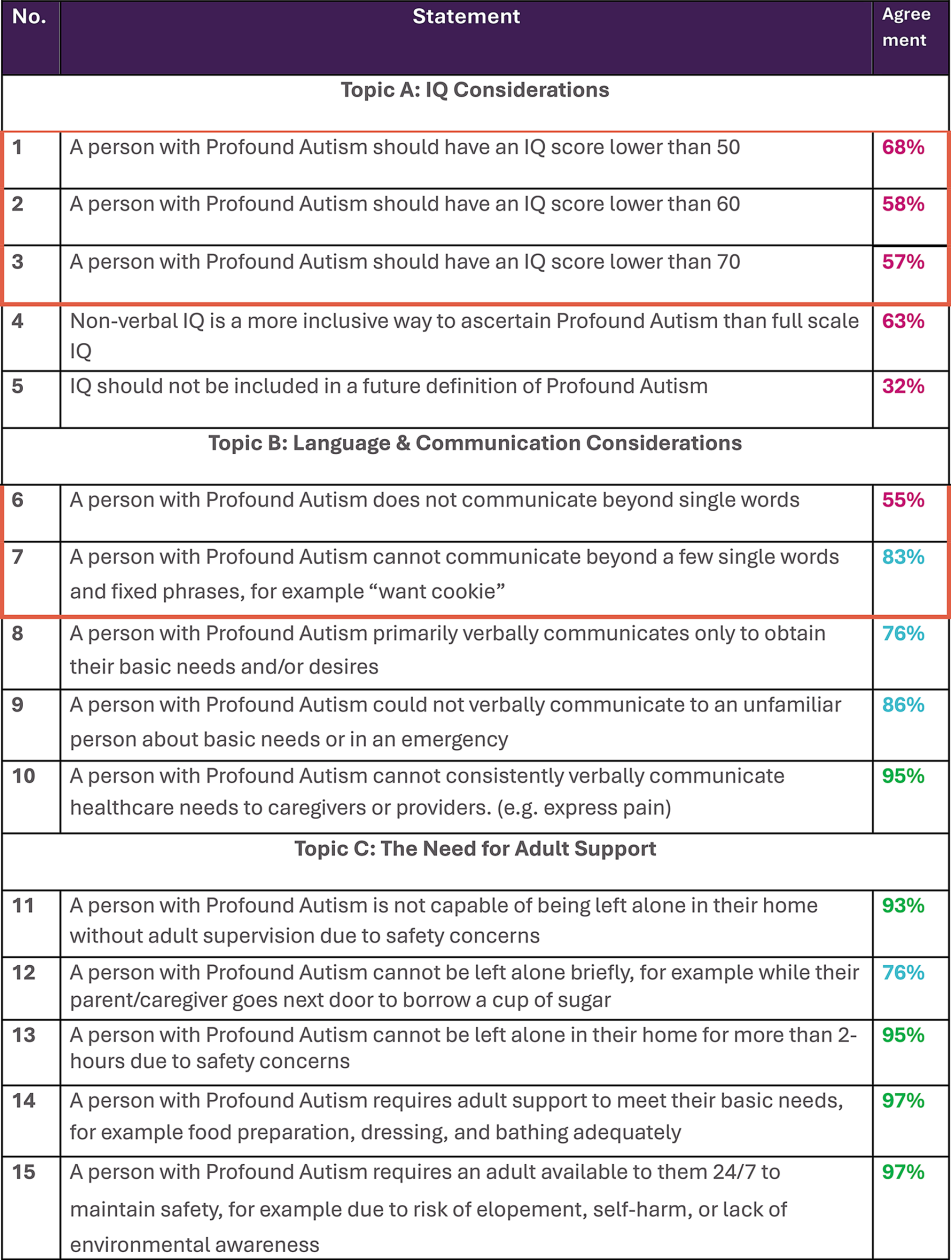

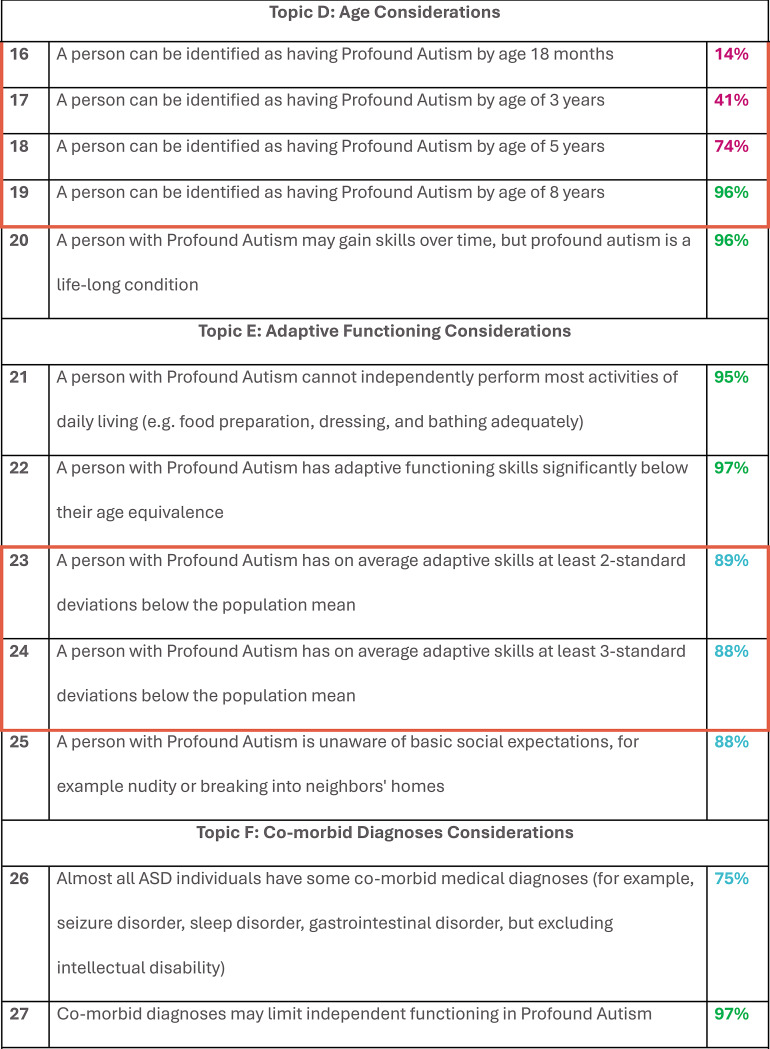

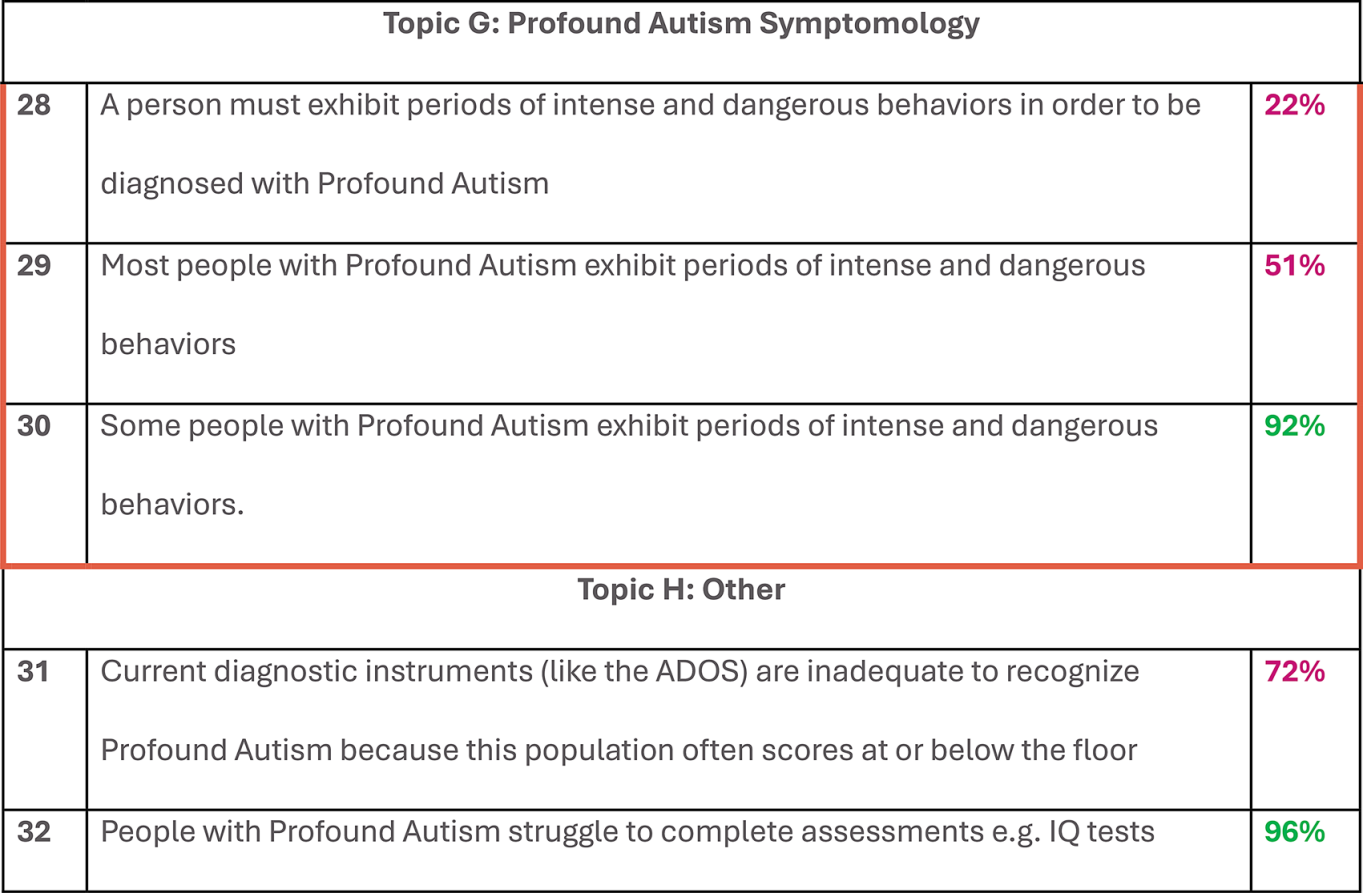
Defined consensus statements and corresponding levels of agreement

Statements which were grouped or paired are indicated with an orange border. Agreement below the consensus threshold (75%) is in pink, agreement which achieved consensus ≥ 75% but < 90% is in blue, agreement ≥ 90% is highlighted in green

## Discussion

Our modified Delphi study led to a consensus research definition of profound autism that states a person with Profound Autism:

- meets the diagnostic criteria for Autism Spectrum Disorder,
- requires adult supervision to ensure physical and mental health, safety, and well-being,
- demonstrates adaptive behavior skills well below age level, with an inability to independently perform most activities of daily living,
- has severely impaired cognitive abilities (reflected by IQ score below 50).

and/or

- does not verbally communicate other than single words or fixed phrases, and
- communicates predominantly to have their basic needs met
- is at least 8 years old (acknowledging that some characteristics consistent with profound autism are evident earlier),

These criteria are neither intermittent nor transient and persist across different environmental settings and situations.

Our study confirms that different stakeholders, drawn from a primarily U.S.-based group of researchers, clinicians and caregivers, widely recognize profound autism. With a more detailed consensus-based definition, researchers can begin filling the many gaps in our knowledge about people with profound autism across the lifespan, to lay the foundation for the development of novel treatments, behavioral interventions, and supports specific to this group, increasing the chance of effective, individualized intervention and improved outcomes. Of importance, we emphasize this as a research definition for profound autism, as there is a need for more information and guidance before it becomes a practical definition utilized in clinical situations. This includes providing guidance in situations where IQ is not regularly assessed, cannot be assessed, or where verbal ability is ambiguous, and the need for guidance around the definition of requiring 24/7 access to an adult for the purposes of health and safety. This may especially be the case in low-resource or rural communities.

This definition aligns closely with the initial definition proposed by the Lancet Commission [2] but offers researchers a more consistent means of operationalizing measurement. Echoing the initial Lancet definition is the requirement for 24/7 access to an adult to maintain health and safety. There was an extremely high level of agreement on this domain, with the broader statements on the need for support to meet basic needs and 24/7 support to maintain safety, achieving 97% agreement. This component of the definition, as simple as it is, achieved such high levels of agreement and garnered so little debate that it suggests that needing access to an adult is a core defining feature of profound autism and is worthy of further assessment and measurement development. Such work will enable us to better understand what this domain represents and how people with this need can be best served and reach their greatest potential by utilizing principles of autonomy, beneficence, and self-direction.

The domain of adaptive functioning, the ability to perform tasks of daily living, such as dressing, bathing, navigating streets, using money, etc., also achieved extremely high agreement across respondents. While a specific cut-off was not identified, the broader concept of a significant gap between the individual’s adaptive functioning level and that expected for age was highly supported. Across the spectrum, adaptive functioning generally lags developmental norms for age, often by several standard deviations. 97% of respondents agreed that adaptive functioning significantly below age-appropriate levels is a necessary element of the PA definition. Some research suggests that adaptive behavior and executive function are more critical than IQ for understanding variance in social and communication domains in those with ASD [22]. Adaptive functioning at an early age is possibly predictive of functioning category at a later age [23], which increases its salience as a component of the PA definition.

There was broad agreement that significant language impairment should be part of the definition. To date, creating a universal definition of minimally verbal in ASD has been challenging [24]. In the surveyed definition of PA, respondents expressed concerns about measuring communication deficits in the context of low IQ. Including being minimally verbal as part of the definition of PA will help to motivate researchers to use non-verbal testing and other strategies proposed within the literature to assess and engage with minimally verbal children [25]. 

There was also a high level of agreement on excluding several domains from the consensus definition. Severe, intense or challenging behaviors are a significant concern with individuals across the spectrum and are particularly prevalent in the profound autism group. Among the many issues raised by these behaviors, an insidious and perhaps most impairing aspect is that the behaviors can result in exclusion from the very activities, experiences, health care, and interventions that the individual needs in order to maintain basic health and connection and reach their full potential. Many caregivers report that it is these behaviors, rather than the autism, that are the primary source of distress and impairment for the individual and family and are of greatest concern [26]. Given this, it was notable that there was a high level of agreement that the presence of these behaviors should not be included in the definition of PA, as there are individuals who do not engage in these behaviors but still need 24/7 support for safety. Identifying what distinguishes this group from the majority that do engage in these behaviors is a critical research opportunity, as clarification of genetic, relational or experiential factors that initiate or accelerate the pathway to these behaviors could lead to broad early intervention guidance that seeks to prevent or ameliorate their occurrence and effects and consequently increase community participation, family and individual well-being, and decrease exclusion from the community, educational settings, intervention programs and healthcare. Co-occurring diagnoses are common in ASD, and some studies have reported higher rates of medical and psychiatric co-occurring disorders in those with characteristics consistent with profound autism, including higher rates of epilepsy, constipation, and ADHD [27, 28]. Despite their prevalence, it was widely agreed among respondents that these co-morbidities should not be included as criteria for profound autism, as there is wide variability in the prevalence of each co-morbidity, and there are individuals in the profound group who lack these co-morbidities.

The area of most significant disagreement and debate was the use of an IQ score cut-off in defining PA. Agreement was greatest for an IQ score lower than 50 (68%), rather than a cut-off of 60 (58%) or 70 (57%), but no cut-off value achieved the consensus threshold. Despite the disagreements, respondents felt IQ should be included (“IQ should not be included” scored only 32% agreement) to enable researchers to use existing databases that include IQs, but not more open-ended questions about support needs. In terms of clinical care and other research, however, respondents expressed concern with both the constraints in access to IQ testing and the validity of testing for those with low cognitive functioning and/or minimal verbal ability. Survey respondents had a very high level of agreement that individuals with PA struggle to complete assessments like IQ tests (96%). Overall, the lack of consensus on an appropriate IQ cut-off reflected philosophical and measurement challenges that have long been debated within the field. an IQ < 50 therefore was included as part of criterion 5 as an “and/or” with minimal verbal ability.

Another area of significant debate was the lower age bound appropriate for application of the term profound autism. Most respondents felt that PA could be identified by age 5 (74%). However, this statement did not reach the consensus threshold, due to the lack of agreement from researchers (57%) compared to caregivers (86%) and autistic individuals (100%). The disagreements among researchers were characterized by three themes. The primary scientific concern was that, in multiple studies, some key elements of the profound autism definition, such as verbal ability or IQ score, are not stable in children under 8 years old [29, 30]. As such, providing a reliable profound autism diagnosis before that age was determined to be unsupported by current data. Participants also noted that round-the-clock adult support is developmentally appropriate for children under the age of 8. A third theme of concern was if profound autism was diagnosed in young children, health insurance companies or policy makers might utilize the term to limit access to more intensive or particular interventions, similar to how some have misused the ADOS and other measures as requirements for establishing an autism diagnosis, which creates barriers to diagnosis and subsequent services [31]. As there currently are no measures that have been shown to reliably predict later profound autism status in those under 8 years old, the consensus was to constrain the definition to individuals 8 and older. This gap represents an important future research opportunity.

Strengths of the study include an evidence-based methodology for establishing scientific consensus, a reasonable response rate, a range of participants including researchers, clinicians, caregivers, autistic individuals, and others, and a result based on broad agreement above the a priori threshold for the specified domains. Limitations of this study include the small proportion of participants who self-identified as autistic, the fact that most participants were from the US and that several participants acknowledged they may have answered differently if better measures or data for challenging areas, such as IQ or age of reliable symptom ascertainment, were available in the field. A major challenge, and a source of debate among respondents, is how to utilize existing data when applying a new definition, which may not be well-measured in current datasets. While new measures and empirical investigation of definitional domains are essential in the future, researchers, caregivers and others were understandably concerned about ensuring that existing data, such as IQ scores or adaptive functioning scores, could be effectively utilized for research or clinical needs when considering profound autism.

Our study provides several clear foci for future research to further operationalize and better understand the definition, measure key domains not well-represented by current measures, and answer critical questions, such as the age at which symptoms of profound autism are reliably predictive of future group inclusion. Needing 24/7 access to an adult to ensure safety and well-being is a concept that is not well-represented by current measures and presents an opportunity for both reliable measure development and parsing the essential and predictive elements captured by this concept. Notably, caregivers of autistic children and adults who participated in the study were in 97% agreement as to the critical nature of this domain, suggesting it captures something essential for the concept and experience of PA. Valid and reliable measurement of IQ in individuals with significant cognitive and/or language impairments remains a major challenge for the field and is also an area for further measure development. Equally important is the opportunity to explore the concurrent validity of other more accessible measures that could adequately indicate or represent level of cognitive impairment. Given the barriers to obtaining valid IQ scores using current measures, respondents engaged in the most debate about whether to include IQ in the definition of profound autism, and future research on representation with and without IQ measurement would be instructive.

Another area that the definition of profound autism highlights as needing further development is efficient, quantitative measurement of verbal ability across the full spectrum. Currently studies use a wide range of measures, which inhibits progress and focus on this primary area of functioning [10]. Nevertheless, among researchers who focus on minimally verbal autism, there is growing agreement that natural language samples represent the best approach to capturing the communicative abilities, including speech and language in this population [32]. 

A key area for further research is identifying at what age characteristics appear that are predictive of meeting the definition of profound autism. Establishing this could widen the age range of research participation and enhance the ability to provide focused and possibly more intensive early intervention for those with the highest likelihood of future impairment, which is a critical need in the field to inform decisions on dose, duration, and cost of early intervention for a particular individual. It is also critically important, given our study’s determination that the need for adult support is a core concept of profound autism, to develop measures with greater specificity for this domain and to parse the items that may carry the most variance, such as the ability to communicate in an emergency [33]. Finally, the development of sensitive and specific screeners for profound autism across the lifespan would benefit both research and clinical settings, allowing rapid identification of at-risk individuals, potentially increasing recruitment efficiency and participation in research, and highlighting critical domains for individualizing treatment.

## Limitations

Despite the rigor of the process, there are limitations to the existing study design. There is geographic and cultural variation in diagnosis and access to services and varying tools to assess intellectual disability measurements which may make an IQ measurement difficult, especially in those who are profoundly affected. We agree that cultural and contextual factors might influence the recognition of features of profound autism, however, we also believe the concept of profound autism applies across the world. The US-based Steering Committee invited a number of clinicians and stakeholders from across the world, but ultimately those that responded were primarily from within the US.

There was also limited demographic information of respondents collected during the survey, although, participants were invited based on their recognized expertise, geographic location, professional roles, caregiving experience, advocacy involvement, or other relevant experience related to profound autism. The absence of more detailed demographic information limits the ability to independently assess the diversity of perspectives represented and should be considered a limitation of the study and we hope future research extends or replicates these findings.

## Conclusions

A large and varied group of researchers, clinicians, caregivers, and autistic individuals agreed that Profound Autism is a meaningful term and reached consensus above the a priori specified level on key components of a research definition. The highest levels of agreement were reached on the requirement of 24/7 access to an adult for health and safety, as well as significant impairment in adaptive functioning. The consensus definition creates the opportunity to compare datasets and health records and to spur the creation of new measures, both of which will enable an increased focus on the research questions and clinical needs of an unresearched and underserved portion of the autism spectrum. As there is evidence that the outcomes, needs, interventions, and supports for those with Profound Autism are distinct, it logically follows that clarity in identification increases the opportunity to improve those areas for both those with profound autism and those without.

## Supplementary Information

Below is the link to the electronic supplementary material.

Supplementary Material 1

Supplementary Material 2

## Data Availability

The dataset supporting the conclusions of this article may be available following a request to the corresponding author.

## Abbreviations

ADHD: Attention Deficit Hyperactivity Disorder
ADOS: Autism Diagnostic Observation Scale
ASD: Autism Spectrum Disorder
DSM: Diagnostic and Statistical Manual for Mental Disorders
IDD: Intellectual Disability
IQ: Intellectual Quotient
NICHD: National Institute of Child Health and Human Development
PA: Profound Autism
SC: Steering Committee
