# Supplementary material for: Developing a consensus research definition for profound autism using a modified Delphi method
Source: Mol Autism. 2026 Jun 30;17:28. doi: 10.1186/s13229-026-00727-y (PMC13326320; doi:10.1186/s13229-026-00727-y)
Supplement: Supplementary file 1 — Supplementary Material 1 [file 13229_2026_727_MOESM1_ESM.docx]

**SUPPLEMENTARY INFORMATION**

**Supplementary Figures**

**Figure S1.** The number of round one survey respondents per role.

**Figure S2.** The number of round one survey respondents by region.

**Figure S3.** The number of round two survey respondents per role.

**Figure S4.** The number of round two survey respondents by region.
