# Supplementary material for: Developing a consensus research definition for profound autism using a modified Delphi method
Source: Mol Autism. 2026 Jun 30;17:28. doi: 10.1186/s13229-026-00727-y (PMC13326320; doi:10.1186/s13229-026-00727-y)
Supplement: Supplementary file 2 — Supplementary Material 2 [file 13229_2026_727_MOESM2_ESM.docx]

**Supplementary Table 1.** Each element of the initially developed definition of profound autism and the summarized qualitative feedback from the second round of Delphi survey, grouped by each variable of the proposed definition.

| **Criterion** | **Proposed Definition** | **Summarized Feedback** |
| --- | --- | --- |
| To be diagnosed with profound autism an individual: | |  |
| **1. Meets diagnostic criteria** | Meets the diagnostic criteria for Autism Spectrum Disorder. | N/A |
| **2. Age** | Is at least 8 years old, acknowledging that some characteristics consistent with profound autism are evident earlier. | - Several respondents were concerned that the age threshold of 8 years may restrict research on younger children who already exhibit profound autism characteristics. - Some suggested lowering the age to 6 years, while others proposed an even lower threshold (4 years) to allow for earlier identification and research opportunities. - There were concerns that this criterion could exclude younger children from funding and research efforts, particularly in early intervention and therapeutics. |
| **3. Adult Supervision** | Requires adult supervision, exceeding age-appropriate levels, to ensure safety (due to risks such as elopement, injury, or lack of environmental awareness). | - It was believed there needed to be more clarification of whether supervision was constant or only in specific circumstances. - There were concerns some individuals may not exhibit safety risk but still require intensive support. |
| **4. Adaptive Functioning Skills** | Demonstrates adaptive functioning skills significantly below age level, with an inability to independently perform most activities of daily living (e.g. bathing, food preparation, dressing) | - There was concern the words “significantly” and “most” were too vague. - Comments linked to criterion 3 regarding levels of support/supervision |
| **5a. IQ** | Severely impaired cognitive abilities, reflected by IQ under 50 | - Many respondents questioned the fixed IQ cutoff of 50, noting that:   - IQ scores in autistic individuals, especially those who are minimally verbal, are often unreliable and variable over time.   - Requiring an IQ score could create barriers to diagnosis due to disparities in access to testing. - Some preferred a functional assessment approach over a strict IQ threshold. - A suggested revision was: *“Severely impaired cognitive abilities, typically reflected by IQ under 50 (if available).”* |
|  | **AND/OR** |  |
| **5b. Verbal Communication** | Generally, does not verbally communicate other than single words or fixed phrases and communicates predominantly only to have their basic needs met. | - Concerns were raised about the wording of “fixed phrases”, which could be unclear (e.g., does this include echolalia or scripting?). - Some argued that verbal ability alone should not define profound autism, as some individuals with higher IQs still have severe communication impairments. - A recommended revision was to focus on overall communication deficits rather than specific phrasing. |
| **6. Traits are Constant and Enduring** | These characteristics endure across time, environmental settings and situations. They are neither intermittent nor transient. | - Some respondents were concerned that the phrase “enduring across time” might not allow for developmental change. - Others pointed out that individuals may meet the criteria at one stage but improve over time, leading to concerns about flexibility in applying the definition. |
